# Association of Prenatal Sugar Consumption with Newborn Brain Tissue Organization

**DOI:** 10.3390/nu13072435

**Published:** 2021-07-16

**Authors:** Paige K. Berger, Catherine Monk, Ravi Bansal, Siddhant Sawardekar, Michael I. Goran, Bradley S. Peterson

**Affiliations:** 1 Department of Pediatrics, The Saban Research Institute, Children’s Hospital Los Angeles, Los Angeles, CA 90027, USA; rabansal@chla.usc.edu (R.B.); ssawardekar@chla.usc.edu (S.S.); bpeterson@chla.usc.edu (B.S.P.); 2 Departments of Obstetrics and Gynecology and Psychiatry, Columbia University Medical Center, New York State Psychiatric Institute, New York, NY 10032, USA; cem31@cumc.columbia.edu

**Keywords:** pregnancy, infant, added sugar, brain development, magnetic resonance imaging

## Abstract

Animal studies have shown that exposure to excess sugar during the prenatal and postnatal periods may alter early brain structure in rat pups. However, evidence in humans is lacking. The aim of this study was to determine associations of maternal total and added sugar intake in pregnancy with early brain tissue organization in infants. Adolescent mothers (*n* = 41) were recruited during pregnancy and completed 24 h dietary recalls during the second trimester. Diffusion tensor imaging was performed on infants using a 3.0 Tesla Magnetic Resonance Imaging Scanner at 3 weeks. Maps of fractional anisotropy (FA) and mean diffusivity (MD) were constructed. A multiple linear regression was used to examine voxel-wise associations across the brain. Adjusting for postmenstrual age, sex, birth weight, and total energy intake revealed that maternal total and added sugar consumption were associated inversely and diffusely with infant MD values, not FA values. Inverse associations were distributed throughout all of the cortical mantle, including the posterior periphery (Bs = −6.78 to −0.57, Ps < 0.001) and frontal lobe (Bs = −4.72 to −0.77, Ps ≤ 0.002). Our findings suggest that maternal total and added sugar intake during the second trimester are significantly associated with features of brain tissue organization in infants, the foundation for future functional outcomes.

## 1. Introduction

Dietary sugar, including added sugar, has become pervasive in the current food environment [1,2]. This is of concern, as dietary sugar consumption can contribute to the progression of chronic health conditions [3]. For example, it is well-documented that excessive dietary sugar intake, both in early and later life, is associated with obesity [4,5,6,7], type-2 diabetes [8,9], and cardiovascular disease [10,11]. Emergent evidence suggests that it is also associated with altered brain and behavioral functioning, ranging from poor self-regulation of food intake [12,13] to impaired cognitive capacities [14,15], and is observed in humans as early as mid-childhood [16]. It is therefore of interest to assess how early exposure to dietary sugar influences brain-related outcomes during critical windows of growth and development, when the fundamental features of brain tissue organization are established [17].

Our research team has particular interest in the influence of acute exposure to dietary sugar early in life on neurodevelopmental outcomes later in life. To date, our work has focused on the period of exclusive breast milk feeding. We have shown that maternal consumption of sugar-sweetened beverages made with high-fructose corn syrup increased fructose in breast milk during early lactation [18]. Moreover, maternal consumption of sugar-sweetened beverages during early lactation was associated inversely with child cognitive performance at 2 years of age [19]. While these results indicate that maternally consumed dietary sugar could conceivably be transmitted to the nursing infant through breast milk [18,20,21], maternal intake of dietary sugar during early lactation may represent enduring consumption patterns persisting from pregnancy, when exposure could influence later neurodevelopmental outcomes of the offspring [16,19].

It is plausible that exposure to any excess dietary sugar influences early brain tissue organization even before birth [17]. Maternally consumed dietary sugar has a direct line of transmission to the developing fetal brain, and the metabolism of dietary sugar may adversely affect the formation of early neural networks [17]. Indeed, in animal models of exposure, rat dams fed a sugar-sweetened solution bore pups expressing increased levels of inflammatory mediators in the hippocampus [22], which in turn was associated with poorer learning and memory [14,22]. Moreover, rodents fed a sugar-sweetened solution had increased expression of oxidative stress mediators in the frontal cortex [23], as well as lower levels of proteins that regulate early brain tissue organization and plasticity [23,24]. Taken together, these findings suggest that fetal exposure to dietary sugar may irreversibly disrupt the formation of neural networks during specific temporal windows of fetal growth, when the brain is developing most rapidly and all the fundamental architectures of the brain are established.

The aim of this study was to use Diffusion Tensor Imaging (DTI) to assess the associations of maternal dietary sugar consumption during the second trimester of pregnancy with early brain tissue organization of infants at three postnatal weeks. DTI is a Magnetic Resonance Imaging (MRI) modality that measures the diffusion of water in the brain, which is determined by characteristics of tissue microstructure.

## 2. Materials and Methods

### 2.1. Subjects

For this prospective cohort study, participants were 72 adolescent females recruited during pregnancy from the Departments of Obstetrics and Gynecology at Columbia University Medical Center (CUMC) and Weill Cornell Medical College. Mothers were included if they: (1) received routine prenatal care; (2) had no major health problems at time of recruitment; (3) were able to read English at a 5th grade level; and (4) were 14 to 19 years of age at time of delivery. Participants in this age group are classified as adolescents, who tend to consume more added sugar than any other age group [25,26]. Mothers were excluded for use of recreational drugs, tobacco, alcohol, or psychotropic medications [27,28]. The Institutional Review Board at Columbia University Medical Center/New York State Psychiatric Institute approved all study procedures. Participants provided written informed consent prior to data collection [27].

### 2.2. Study Design

Participant health-related information was abstracted from the medical record and included family health history, maternal health history, and infant characteristics at birth [28]. Participants completed three visits during pregnancy, once in each trimester, though the present study is focused on findings from the second trimester only. The second trimester was selected for several reasons: (1) the number of neurons born in the brain peaks over the course of the second trimester, and it is therefore a critical temporal window for early brain tissue organization, neural circuit formation, and influence from exogenous exposures [17]; (2) maternal consumption of total sugar correlated most highly with added sugar during the second trimester of pregnancy (*r* = 0.89, *p* < 0.01) compared to the first trimester and third trimester. Infants underwent MRI scanning within the first few weeks of birth [28].

### 2.3. Dietary Intake of Mothers

At the second trimester visit, maternal dietary intake was determined using the Automated Self-Administered 24-Hour (ASA-24) Dietary Assessment Tool from the National Cancer Institute. ASA-24 is a web-based questionnaire that collects food and beverage intake from the preceding 24 h with the use of detailed probes and scale images of portion sizes to assist in estimation of serving sizes. ASA-24 then determines the relative amounts of nutrients consumed from the preceding 24 h using three databases: (1) US Department of Agriculture (USDA) Food and Nutrient Database for Dietary Surveys; (2) USDA MyPyramid Equivalents Database (MPED); and (3) USDA Center for Nutrition Policy and Promotion’s MPED Addendum [28,29]. It is important to note that ASA-24 does not account for naturally occurring free sugar, i.e., not bound to fiber, in its assessment of added sugar, despite having the same metabolic fate [19,30,31]. Therefore, we explored associations of both total sugar and added sugar with DTI-based outcomes in separate analytical models, as total sugar includes free sugar derived from both added sugar and naturally occurring sources.

### 2.4. MRI Scanning of Infants

#### 2.4.1. MRI Scanning Procedures

Infants completed MRI scanning procedures within 3 weeks of birth. Infants were fed, swaddled, and acclimated to the MRI scanning environment. Infants were allowed time to fall asleep on the MRI scanning bed before the start of each pulse sequence, without the use of sedation. Foam and wax ear plugs and ear shields were also applied to dampen scanning sounds [27]. MRI-compatible EKG leads were fixed on the infant’s chest and a pulse oximetry sensor was fixed on the infant’s toe. Heart rate and oxygen saturation were monitored throughout MRI scanning [27]. Ambient noise with recordings of scanning sounds that were played before each pulse sequence to reduce alerting to the start of each sequence [27].

Infant brain images were acquired using a 3.0 Tesla General Electric Signa MRI scanner with an 8-channel head coil. DTI was then used to assess the direction and rate of diffusing water, which is influenced by early brain tissue organization (e.g., nerve fiber orientation, axonal density, degree of myelination), and indexed by fractional anisotropy (FA), mean diffusivity (MD), axial diffusivity (AD), and radial diffusivity (RD). Briefly, FA is a measure of the preferential directional diffusion of water and reflects, among other things, the degree of myelination [32]. MD is an overall measure of the diffusion of water without reference to direction: it comprises AD and RD, which provide indices of diffusion, respectively, either parallel or perpendicular to the long axis of nerve fiber bundles [17,32]. As FA reflects in part the degree of myelination, which may not yet be evident at 3 postnatal weeks, MD may be a more robust indication of infant brain tissue organization at this stage of development, when it likely references overall neuronal cell density and other macromolecular constraints on the diffusion of water [33,34].

DTI data were acquired in axial oblique slices parallel to the anterior–posterior commissure line using a single-shot echo planar DTI imaging sequence, with repetition time = 13,925 ms, echo time = 74 ms, field of view = 19 × 19 cm^2^, flip = 90°, acquisition matrix = 132 × 128 (acceleration factor = 2) zero-padded to 256 × 256, for 60 oblique axial contiguous slices positioned parallel to the anterior–posterior commissure line, slice thickness = 2.0 mm [28]. We acquired 3 baseline images with b = 0 s/mm^2^ and 11 diffusion-weighted images at b = 600 s/mm^2^, with diffusion gradients applied in 11 directions sampling 3D space uniformly [28].

#### 2.4.2. MRI Data Processing

We corrected magnetic field inhomogeneities, eddy-current distortions, and head motion using the FSL toolbox [35]. We then used a Levenburg–Marquardt algorithm to achieve a robust non-linear least-squares fit in estimating the diffusion tensor at each voxel, fitting an ellipsoid to the diffusion-weighted imaging data along the 11 gradient directions and 3 baseline images while constraining the diffusion tensor to be positive definite [36]. RD maps generated from the diffusion tensor model were then spatially normalized to the template brain using a rigid body similarity transformation, followed by a non-linear warping using a method based on fluid dynamics [37]. We also calculated the calculated scalar indices for the tensor at each voxel. For a diffusion tensor D with eigenvalues $\left(\lambda_{1},\lambda_{2},\lambda_{3}\right)$ ordered such that $\lambda_{1}>\lambda_{2}\geq \lambda_{3}$ [38], $FA=\frac{\sqrt{{\left(\lambda_{1}-\hat{\lambda }\right)}^{2}+{\left(\lambda_{2}-\hat{\lambda }\right)}^{2}+{\left(\lambda_{3}-\hat{\lambda }\right)}^{2}}}{\sqrt{2\left(\lambda_{1}^{2}+\lambda_{2}^{2}+\lambda_{3}^{2}\right)}}$, $MD=\hat{\lambda }=\frac{\lambda_{1}+\lambda_{2}+\lambda_{3}}{3}$, $RD=\frac{\lambda_{2}+\lambda_{3}}{2}$, and *AD* = $\lambda_{1}$.

We employed a 2-step procedure to select a template brain to ensure that findings were not unduly influenced by selection of a non-representative template. First, using the T2-weighted anatomical images for each infant, we identified, as a preliminary template, the brain of one infant whose postmenstrual age and overall brain size were nearest the group averages. Postmenstrual age at the time of MRI scan is defined as the time that elapsed between the mother’s last menstrual period and birth of the infant, also known as gestational age at birth, plus chronological age from birth to the time of MRI scan [39]. The brains for all remaining unexposed infants in the sample were coregistered to that preliminary template, and then the distance of each point on the surface of each brain was measured from the corresponding point on the preliminary template surface. The brain for which all points across its surface were closest (in the least squares sense) to the average of the distances across those points for the entire sample was selected as the final template, thereby yielding a template brain that was specific to and morphologically most representative of all infant brains in this cohort.

### 2.5. Statistical Analysis

Descriptive statistics are presented as mean ± standard deviation (SD) for continuous variables and as frequency (percentage) for categorical variables. Normal distribution and homogeneity of variances were confirmed by Shapiro–Wilk W and Levene’s tests, respectively. We also calculated Cook’s Distances (*D_i_*) for maternal total and added sugar values and plotted individual data points to find potential influential outliers. An influential outlier was defined as a data point with a *D_i_* value > 1 or with a *D_i_* value that stands out from the rest [40]. We assessed the significance of the correlation coefficient of maternal consumption of total sugar during the second trimester with infant FA and MD values at 3 postnatal weeks. We tested the correlations of infant AD and RD values as post hoc analyses to aid our biological interpretations for MD findings. We tested these associations using multiple linear regression applied voxel-wise throughout the brain. In addition, we assessed the association of maternal consumption of added sugar in the second trimester with DTI-based measures. Postmenstrual age at the time of MRI scan, infant birth weight, infant sex, and total energy intake in the second trimester were included as covariates in all analyses because infant characteristics [41] and variations in caloric intake [28,42] could both influence DTI measures. All covariates were continuous variables apart from infant sex, which was a categorical variable [43]. *p*-values for regressions modeling maternal total and added sugar consumption were corrected for the number of statistical comparisons using the Benjamini–Yekutieli procedure for false discovery rate [44]. Corrected *p*-values were color-coded and displayed on the template brain. All imaging analyses were conducted using software developed in-house.

## 3. Results

Characteristics of the mother–infant dyads are shown in Table 1. Of the 72 adolescent mothers recruited, analyses were limited to the 41 participants whose infants had usable MRI data within 3 weeks of birth (Figure S1). Mothers were predominantly Hispanic. Infants were born at term with a normal birth weight and had similar proportions of males and females. Mothers consumed 2549 ± 1151 kcal per day during the second trimester of pregnancy, of which 26.7% was total sugar and 16.6% was added sugar. Based on these values, the mean intake of added sugars among mothers exceeded the recommendation of the Dietary Guidelines for Americans to limit consumption of added sugar to less than 10% of total energy intake per day. In addition, mothers consumed 2458 ± 1049 kcal per day during the third trimester of pregnancy, of which 24.2% was total sugar and 15.5% was added sugar. Maternal intake of kcal per day (*r* = 0.51, *p* < 0.001) and added sugar per day (*r* = 0.34, *p* = 0.012) during the second trimester of pregnancy were positively associated with reported intake during the third trimester.

**Figure 1:**
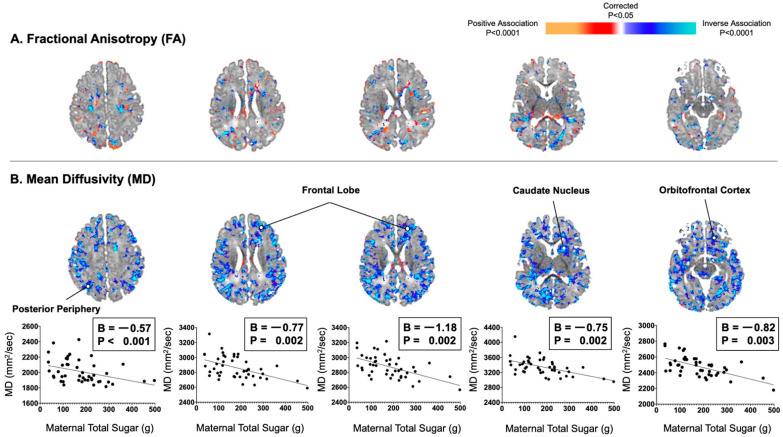
Maternal consumption of total sugar in the second trimester correlated inversely and diffusely with infant MD values, not FA values. (**A**) Maternal total sugar in pregnancy was not convincingly associated with infant FA values. (**B**) Maternal total sugar in pregnancy was associated with infant MD values in locations similar to those observed with maternal added sugar in pregnancy. Associations were distributed evenly throughout all of the cortical mantle or adjacent axons of future white matter.

Maternal consumption of total sugar did not correlate significantly with infant FA values in a consistent or convincing way anywhere in the brain. Maternal consumption of total sugar significantly correlated with infant MD values inversely and diffusely throughout the brain (Figure 1). These inverse associations were distributed evenly throughout all of the cortical mantle or adjacent axons of future white matter, including the posterior periphery (*B* = −0.57, *p* < 0.001) and frontal lobe (Bs = −1.18 to −0.77, Ps = 0.002).

**Figure 2:**
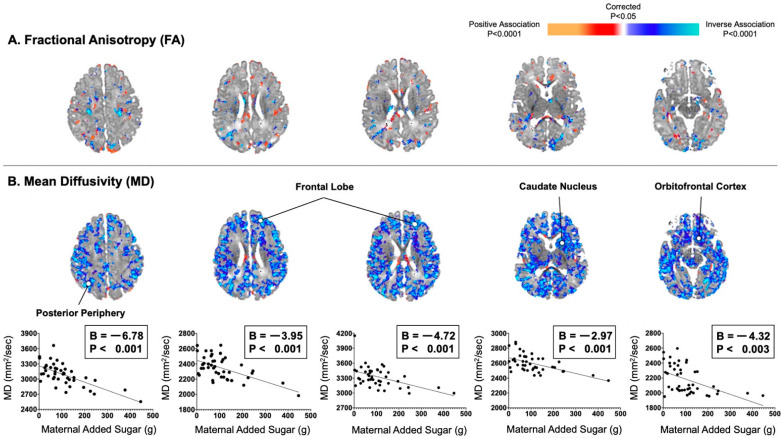
Maternal consumption of added sugar in the second trimester correlated inversely and diffusely with infant MD values, not FA values. (**A**) Maternal added sugar in pregnancy was not convincingly associated with infant FA values, which typically reflects degree of myelination in areas of future white matter. (**B**) Maternal added sugar in pregnancy was associated with infant MD values evenly throughout all of the cortical mantle or adjacent axons of future white matter.

In addition, associations of maternal consumption of added sugar with DTI-based measures were similar to those observed for maternal total sugar consumption (Figure 2). For example, maternal consumption of added sugar did significantly correlate with infant MD values inversely and diffusely in the posterior periphery (*B* = −6.78, *p* < 0.001) and frontal lobe (Bs = −4.72 to −3.95, Ps < 0.001), as well as in the orbitofrontal cortex (*B* = −4.32, *p* = 0.003). In post hoc analyses, maternal consumption of total and added sugar were also found to be associated inversely and diffusely with infant AD and RD values in locations similar to those observed for MD values (Figure S2).

## 4. Discussion

In this study of adolescent mothers, we examined associations of prenatal consumption of dietary sugar during the second trimester of pregnancy with DTI-based measures of infant brain tissue organization, the structural basis for future cognitive, emotional, and behavioral functioning. We found that prenatal consumption of total and added sugar in mothers was inversely and diffusely associated with DTI-derived MD values, but not FA values. While the findings exhibited no clear preferential clustering in specific brain regions, robust associations were observed in the cerebral periphery that likely included cortical gray matter and immediately adjacent axonal projections. Post hoc analyses revealed that prenatal intake of total and added sugar also correlated inversely with both AD and RD values, which further indicates that exposure to total and added sugar affects components of cortical gray matter, including the formation of dendrites and synapses. Overall, our DTI-based findings lend insight into features of brain tissue organization in infants that may be most influenced by exposure to dietary sugar in the second trimester of pregnancy.

Chronic consumption of dietary sugar, specifically as added sugar, has been identified as a potential contributor to mental health conditions, including anxiety, depression, and cognitive decline in adolescents and adults [45,46,47]. Nevertheless, few studies have examined the influence of total and added sugar earlier in life, when the brain is most vulnerable to nutritional insults that may affect future neurodevelopmental outcomes [16,22]. Research on the prenatal and early postnatal periods is perhaps most pressing, as dietary sugar can conceivably be transmitted from the maternal diet to the developing fetus or nursing infant. While limited, data currently indicate that mothers who consume added sugar while pregnant have offspring with lower intelligence test scores: one prior study reported that every one-serving increase in sugar-sweetened beverages consumed in the second trimester of pregnancy was accompanied by a 1.2 point decrement in verbal test scores in children [16]. Moreover, we found that every additional serving of sugar-sweetened beverages consumed in early lactation was associated with a reduction in cognitive scores by age 2 years [19]. It is therefore plausible that added sugar in particular interferes with the earliest features of brain tissue organization that form the structural basis for those future neurodevelopmental outcomes. These findings may be used to guide the latest nutrition recommendations for mothers during pregnancy and lactation, and they may inform the development and design of nutrition interventions to optimize the development of cognitive capacities during infancy.

In this study, we examined the influence of total and added sugar on features of brain tissue organization with the use of DTI, an MRI modality in which the movement of water in the brain is modulated by micro-structural characteristics of future white matter and cortical gray matter. DTI studies have found that MD values, which measure the movement of water without reference to direction, tend to increase in cortical gray matter during late gestation, whereas FA values tend to decrease in cortical gray matter during the same time period [48,49]: higher MD values with advancing gestational age may reflect age-related increases in the number and complexity of dendritic arbors, which branch in all directions without directional specificity [17,48,50,51]. It follows that greater dendritic arborization will increase diffusion of water in all directions and thereby without directional specificity, which will be reflected not only in higher infant MD values, but also in higher AD and RD values. In contrast, we found that lower MD values in infants were associated with greater prenatal consumption of total and added sugar in mothers. We also found that associations were distributed similarly between AD and RD values, which indicates that exposure to total and added sugar may be more influential on processes that underlie the formation of dendritic arbors as opposed to early myelination. The absence of significant associations between FA values in infants and prenatal consumption of dietary sugar in mothers provides additional evidence that effects of added sugar are unlikely to be solely or even primarily on the myelination of axons within future white matter.

Reduced dendritic arborization among infants exposed to total and added sugar during gestation is a potential concern: dendritic arbors provide the structural support for synaptogenesis, which influences the formation of neural circuits and, consequently, information processing in the brain, including learning and memory. While the exact mechanism through which increased exposure to added sugar would reduce dendritic arborization is not known, animal models have shown that rodents fed a sugar-sweetened solution exhibit poorer performance on learning and memory tasks relative to controls. These findings have been attributed in part to elevated production of inflammatory mediators and reduced production of brain-derived neurotrophic factor (BDNF) in the hippocampus and frontal cortex [14,23,24]. It has been postulated that added sugar-induced expression of inflammatory cytokine IL-1β blocks the expression of BDNF, which had downstream effects on cyclic AMP-response element-binding protein (CREB), a critical molecule for the transcriptional regulation of dendritic complexity [14,23,52,53,54]. In vitro studies have also shown that reduced CREB expression impedes dendritic growth and arborization of newborn hippocampal neurons [55,56]. These findings suggest that prenatal exposure to high amounts of added sugar may ultimately interfere in the production of proteins needed for the construction of neural networks. Moreover, there is evidence of additional mechanisms: these include the effects of exposure to added sugar on DNA methylation of genes that may contribute to the assembly of new synapses during fetal development [57,58].

This study has several limitations. The prospective observational design cannot be used to establish causality. Randomized controlled trials that manipulate maternal consumption of total and added sugar during pregnancy would be required to yield that level of causal inference. Though multiple covariates were included in our analyses, residual confounding caused by unmeasured factors could influence our findings. Information regarding maternal nutrition was based on self-reports, not direct observation or measurement. It is possible that participants underestimated (or overestimated) their consumption of total energy and dietary sugar per day [31,59], though reported intakes were similar to what has been observed in studies of similar age groups and correlated across trimesters [26,60]. Finally, our findings are also limited to a relatively small sample of adolescent Hispanic mothers in northeastern United States: differences in built environment, food choice, and other characteristics may limit the generalizability of our overall findings.

## 5. Conclusions

In conclusion, our findings revealed that maternal consumption of dietary sugar during the second trimester of pregnancy was associated inversely and diffusely with DTI-derived MD values in infants, a measure that reflects, in part, the anatomical foundation for future neurodevelopmental outcomes [17].

## Figures and Tables

**Table 1:** Characteristics of healthy maternal participants and their infants ^1^.

V	Mean	SD	Minimum	Maximum
Mothers				
Age at delivery (years)	18.2	1.37	14.0	20.0
Prepregnancy BMI (kg/m^2^)	25.2	6.37	14.4	41.2
Vaginal delivery (%)	83			
Hispanic Ethnicity (%)	95			
Total energy per day, second trimester (kcals)	2549	1151	851	6695
Added sugar per day, second trimester (g)	106	91.5	2.03	446
Total sugar per day, second trimester (g)	170	102	36.0	498
Infants				
Male (%)	61			
Postmenstrual age (weeks)	42.6	1.7	38.7	47.0
Chronological age (days)	23.9	13.2	4.00	94.0
Birth weight (g)	3204	456	2466	4380

^1^ Values are mean ± SD or %.

## Data Availability

Data described in the manuscript, code book, and analytic code will be made available upon request pending application and approval from the authors.

## Supplementary Materials

The following are available online at https://www.mdpi.com/article/10.3390/nu13072435/s1, Figure S1: Participant flow chart, Figure S2: Maternal consumption of added sugar in the second trimester correlated inversely and diffusely with infant axial diffusivity (AD) and radial diffusivity (RD) values. (A) Maternal added sugar in pregnancy was associated with infant AD values in locations similar to those observed with infant MD values. (B) Maternal added sugar in pregnancy was also associated with infant RD values in locations similar to those observed with infant MD and AD values throughout the cortical mantle and adjacent axons of future white matter.
