# Supplementary material for: Association of Prenatal Sugar Consumption with Newborn Brain Tissue Organization
Source: Nutrients. 2021 Jul 16;13(7):2435. doi: 10.3390/nu13072435 (PMC8308814; doi:10.3390/nu13072435)
Supplement: Supplementary file 1 [file nutrients-13-02435-s001.zip › nutrients-1192500-SI.pdf]

Association of Prenatal Sugar Consumption with Newborn Brain Tissue Organization

Paige K. Berger [1,*], Catherine Monk [2], Ravi Bansal [1], Siddhant Sawardekar [1], Michael I. Goran[1,*] and Bradley S. Peterson [1]

Supplementary Materials:

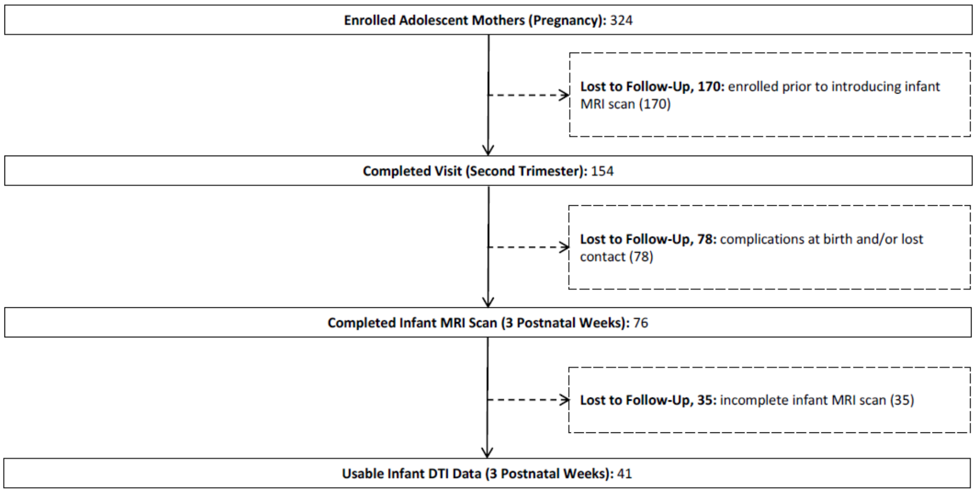

Figure S1. Participant flow chart.

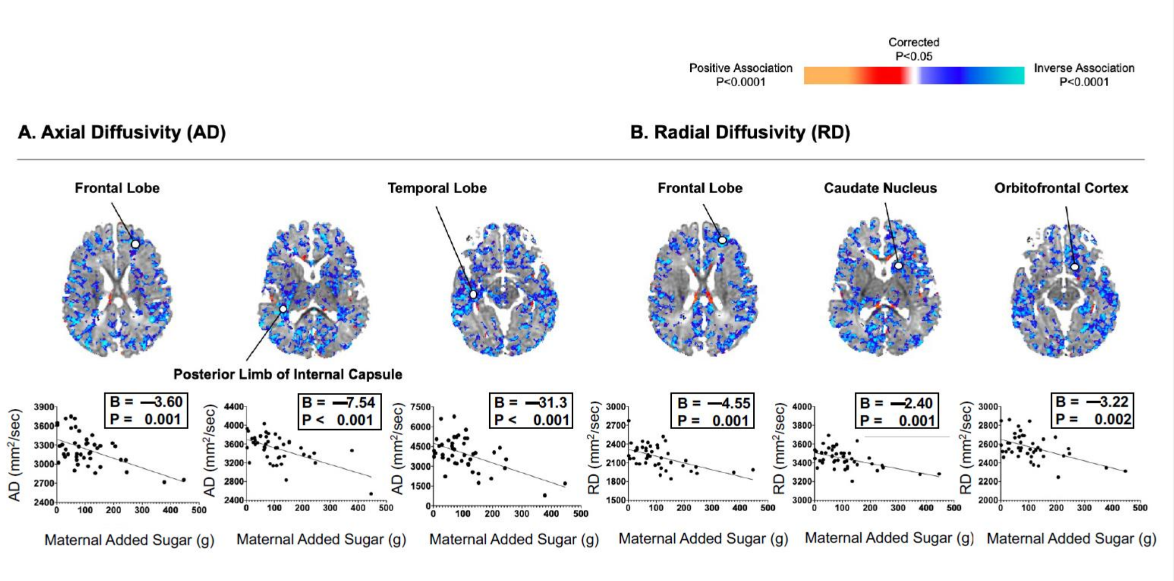

Figure S2. Maternal consumption of added sugar in the second trimester correlated inversely and diffusely with infant axial diffusivity (AD) and radial diffusivity (RD) values. (A) Maternal added sugar in pregnancy was associated with infant AD values in locations similar to those observed with infant MD values. (B) Maternal added sugar in pregnancy was also associated with infant RD values in locations similar to those observed with infant MD and AD values throughout the cortical mantle and adjacent axons of future white matter.
